# SARS-CoV-2 Spike Protein and Mouse Coronavirus Inhibit Biofilm Formation by *Streptococcus pneumoniae* and *Staphylococcus aureus*

**DOI:** 10.3390/ijms23063291

**Published:** 2022-03-18

**Authors:** Mun Fai Loke, Indresh Yadav, Teck Kwang Lim, Johan R. C. van der Maarel, Lok-To Sham, Vincent T. Chow

**Affiliations:** 1 Infectious Diseases Translational Research Program, Department of Microbiology and Immunology, Yong Loo Lin School of Medicine, National University of Singapore, Singapore 117545, Singapore; lokemunfai@u.nus.edu (M.F.L.); lsham@nus.edu.sg (L.-T.S.); 2 Department of Physics, Faculty of Science, National University of Singapore, Singapore 117542, Singapore; iykumarindresh288@gmail.com (I.Y.); johan-maarel@nus.edu.sg (J.R.C.v.d.M.); 3 Protein and Proteomics Centre, Department of Biological Sciences, Faculty of Science, National University of Singapore, Singapore 117558, Singapore; dbslimtk@nus.edu.sg

**Keywords:** *Streptococcus pneumoniae*, *Staphylococcus aureus*, biofilm, COVID-19, SARS-CoV-2, coronavirus, spike protein, S1 and S2 subunits, nucleocapsid, MHV

## Abstract

The presence of co-infections or superinfections with bacterial pathogens in COVID-19 patients is associated with poor outcomes, including increased morbidity and mortality. We hypothesized that SARS-CoV-2 and its components interact with the biofilms generated by commensal bacteria, which may contribute to co-infections. This study employed crystal violet staining and particle-tracking microrheology to characterize the formation of biofilms by *Streptococcus pneumoniae* and *Staphylococcus aureus* that commonly cause secondary bacterial pneumonia. Microrheology analyses suggested that these biofilms were inhomogeneous soft solids, consistent with their dynamic characteristics. Biofilm formation by both bacteria was significantly inhibited by co-incubation with recombinant SARS-CoV-2 spike S1 subunit and both S1 + S2 subunits, but not with S2 extracellular domain nor nucleocapsid protein. Addition of spike S1 and S2 antibodies to spike protein could partially restore bacterial biofilm production. Furthermore, biofilm formation in vitro was also compromised by live murine hepatitis virus, a related beta-coronavirus. Supporting data from LC-MS-based proteomics of spike–biofilm interactions revealed differential expression of proteins involved in quorum sensing and biofilm maturation, such as the AI-2E family transporter and LuxS, a key enzyme for AI-2 biosynthesis. Our findings suggest that these opportunistic pathogens may egress from biofilms to resume a more virulent planktonic lifestyle during coronavirus infections. The dispersion of pathogens from biofilms may culminate in potentially severe secondary infections with poor prognosis. Further detailed investigations are warranted to establish bacterial biofilms as risk factors for secondary pneumonia in COVID-19 patients.

## 1. Introduction

Among COVID-19 patients, the prevalence of co-infection varies in different studies [1]. Well-recognized co-pathogens include bacteria (e.g., *Streptococcus pneumoniae* or *S. pneumoniae*, and *Staphylococcus aureus,* or *S. aureus*); fungi (*Candida* and *Aspergillus* species), and viruses (e.g., influenza and rhinovirus). Notably, in a retrospective multi-center cohort study involving 191 adult in-patients with laboratory-confirmed COVID-19 from Wuhan (China), half of non-survivors (27/54) suffered from secondary infections [2]. In contrast, only one out of 137 survivors (<1%) experienced secondary infection. In another study of patients with COVID-19 pneumonia, nearly half (25/53) of them had additional infection with *Mycoplasma pneumoniae* [3]. One large study of hospitalized COVID-19 patients reported that *S. aureus* and *Haemophilus influenzae* were the most common pathogens causing respiratory co-infections, while *Enterobacteriaceae* and *S. aureus* were most common in secondary respiratory infections [4]. Another report found co-infections between severe acute respiratory syndrome coronavirus 2 (SARS-CoV-2) and other respiratory pathogens to be relatively common [5]. However, the prevalence of bacterial co-infections among COVID-19 patients may be underestimated since broad-spectrum antibiotics were widely prescribed to these patients, as revealed in an international survey involving 23 countries and 82 hospitals [6]. The use of antibiotics may reflect the experiences with bacterial super-infections in influenza that are often caused by *S. pneumoniae* and *S. aureus* [7].

Co-infecting bacterial pathogens constitute a major cause of morbidity and mortality in influenza. The most common co-infecting bacteria are *S. pneumoniae* and *S. aureus*, which account for 35 and 28% of infections, respectively [8]. Asymptomatic nasal carriage of *S. aureus* occurs in about 30% of the human population [9]. *S. pneumoniae* is also able to colonize transiently and asymptomatically in immunocompetent hosts [10]. Although these bacteria are part of the commensal microbiota, a trigger such as a viral infection can generally increase the occurrence of co-infections with potentially serious consequences [11]. Interestingly, co-infection scenarios of influenza viruses with *S. pneumoniae* or *S. aureus* differ in their sequence of infection [12]. Whereas *S. pneumoniae* often causes severe bacterial infections after viral clearance, co-infections with *S. aureus* are more likely to occur concomitantly.

The recognition of SARS-CoV-2 infection is critical as it facilitates the prompt implementation of appropriate infection control measures and antiviral therapy. However, the possibility of co-infections of SARS-CoV-2 with bacterial pathogens should always be considered. Although bacteria do not directly support eukaryotic virus infection, they can promote viral fitness by enhancing virion stability, promoting infection of eukaryotic cells, and increasing co-infection rates. Virus binding of bacteria can also impact bacterial biology, including bacterial adherence to eukaryotic cells. These mutual interactions can also indirectly affect the host response to viral infection. For example, influenza A virus (IAV) can bind directly to both Gram-positive (*S. pneumoniae* and *S. aureus*) as well as Gram-negative bacteria (*H. influenzae*), enhance bacterial adherence to epithelial cells, and increase uptake by macrophages [13,14]. Respiratory syncytial virus (RSV), human parainfluenza virus 3 (HPIV-3) and IAV infections increase bacterial adhesion to primary and immortalized respiratory cell lines. Moreover, RSV and HPIV-3 can increase the expression of known bacterial receptors associated with bacteria–host interactions [15]. Virus–bacteria interactions and their impact on bacterial adherence to host cells during co-infection or secondary infection are likely to be complex and multi-factorial [16]. Bacteria can also adhere to host cells through the formation of biofilm, leading to persistent infections, or the dispersal of biofilm may increase the risk of developing systemic infections. In this study, we hypothesized, investigated, and compared the effects of recombinant SARS-CoV-2 spike (S) and nucleocapsid (N) proteins on biofilm formation by *S. pneumoniae* and *S. aureus* in vitro. To further support our observations, we also analyzed the interactions between bacterial biofilms and live mouse hepatitis virus (MHV), which served as a surrogate model of beta-coronavirus.

## 2. Results

To model bacterial biofilm formation in vitro by two Gram-positive bacteria (*S. pneumoniae* and *S. aureus*) that commonly cause pneumonia, six *S. pneumoniae* and three *S. aureus* strains (Table 1) were initially screened on 96-well plates using a crystal violet assay.

**Figure 1:**
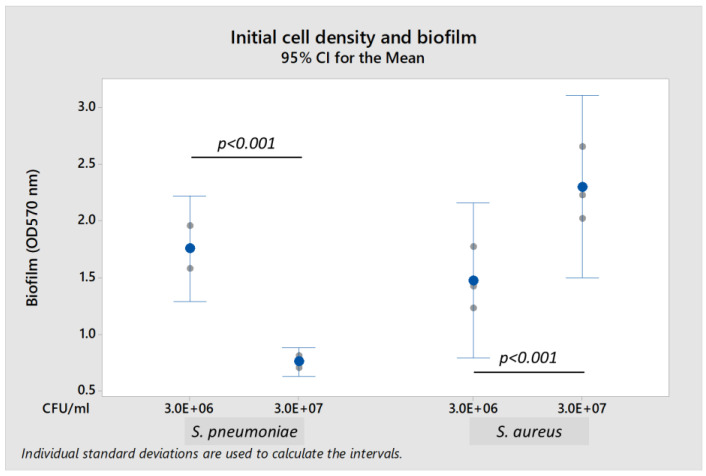
Effect of initial bacterial cell density on biofilm production by *S. pneumoniae* 19F and *S. aureus* A10 strains using the crystal violet assay. Each result was derived from three independent experiments or biological replicates (with each assay performed as technical triplicates). Each small gray datapoint represents the average or mean of technical triplicates, while each blue datapoint depicts the mean of three independent experiments. Intervals represent 95% confidence interval (CI) for the mean. *p*-value < 0.05 was considered statistically significant by the independent samples Mann-Whitney U test.

Both *S. pneumoniae* 19F and *S. aureus* A10 strains formed biofilm when cultured in brain heart infusion (BHI) broth and nutrient broth, respectively. The presence of 0.2% glucose enhanced biofilm formation. Biofilm-forming capability of *S. pneumoniae* 19F and *S. aureus* A10 depended upon the initial bacterial density (Figure 1). *S. pneumoniae* formed biofilm optimally at a lower initial bacterial load of 3.0 × 10^6^ colony-forming units per mL (CFU/mL), but did not form biofilm at higher initial load of 3.0 × 10^7^ CFU/mL (*S. pneumoniae* biofilm was reduced by 56.7% when compared to load of 3.0 × 10^6^ CFU/mL). In contrast, *S. aureus* formed more biofilm when initial bacterial load increased from 3.0 × 10^6^ CFU/mL to 3.0 × 10^7^ CFU/mL (biofilm formation was 160% higher). Thus, subsequent experiments were performed using initial bacterial loads of 3.0 × 10^6^ CFU/mL for *S. pneumoniae* and 3.0 × 10^7^ CFU/mL for *S. aureus* in their respective media supplemented with 0.2% glucose.

To investigate the effects of the SARS-CoV-2 spike (S) protein on bacterial biofilm formation, recombinant S1 subunit, S2 extracellular domain (ECD), and both S1 + S2 subunits were tested on *S. pneumoniae* and *S. aureus*. Another abundant SARS-CoV-2 structural protein, i.e., recombinant nucleocapsid protein (NP) was also investigated to serve as a comparative and reference control.

**Figure 2:**
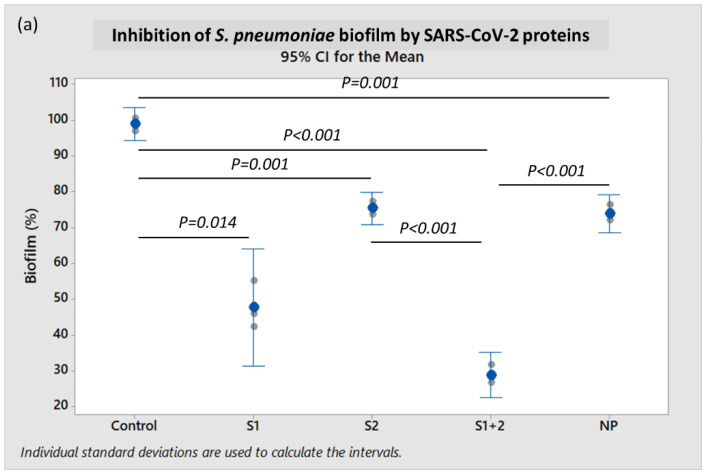

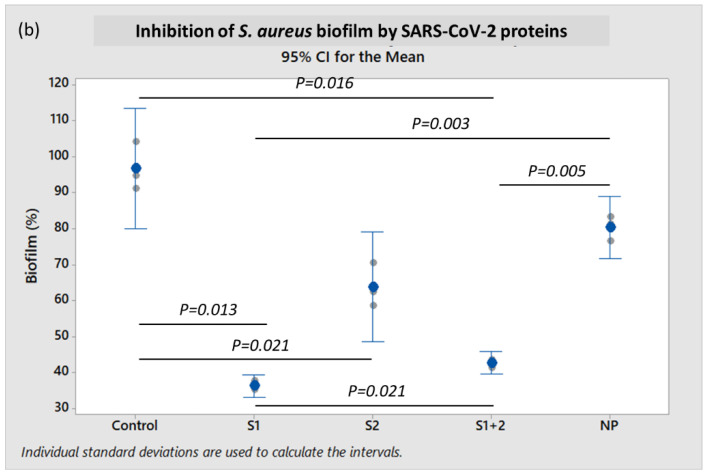
Characterization of biofilm production by (**a**) *S. pneumoniae* 19F and (**b**) *S. aureus* A10 strains, when co-incubated with recombinant SARS-CoV-2 spike S1 subunit, S2 ECD, S1 + S2 subunits, and nucleocapsid protein (NP) using the crystal violet assay. Each result was derived from three independent experiments or biological replicates (with each assay performed as technical triplicates). Each small gray datapoint represents the average or mean of technical triplicates, while each blue datapoint depicts the mean of three independent experiments. Intervals represent 95% CI for the mean. *p*-value < 0.05 was considered statistically significant by one-way ANOVA with Dunnett’s T3 post-hoc test.

When incubated in the presence of 10 pmol/mL of recombinant S1 + S2 subunits at 37 °C for 18 h, the mean *S. pneumoniae* biofilm formation was very significantly diminished when compared to control (*p* < 0.001), and was reduced by 61.0% (*p* < 0.001) when compared to incubation with NP (Figure 2a). When incubated with the S1 subunit alone, *S. pneumoniae* biofilm was reduced by 35.4% when compared to NP, but this reduction was not statistically significant (*p* ≥ 0.05). However, mean *S. pneumoniae* biofilm formation when incubated with S2 ECD alone was also not significantly different from the NP-treated control.

The mean *S. aureus* biofilm formation decreased by 46.8% (*p* = 0.005) in the presence of 10 pmol/mL of both S1 + S2 subunits, and by 54.8% (*p* = 0.003) with S1 subunit alone when compared to incubation with NP (Figure 2b). In contrast, mean *S. aureus* biofilm formation when incubated with S2 ECD did not exhibit a statistically significant difference when compared to NP (*p* ≥ 0.05).

**Figure 3:**
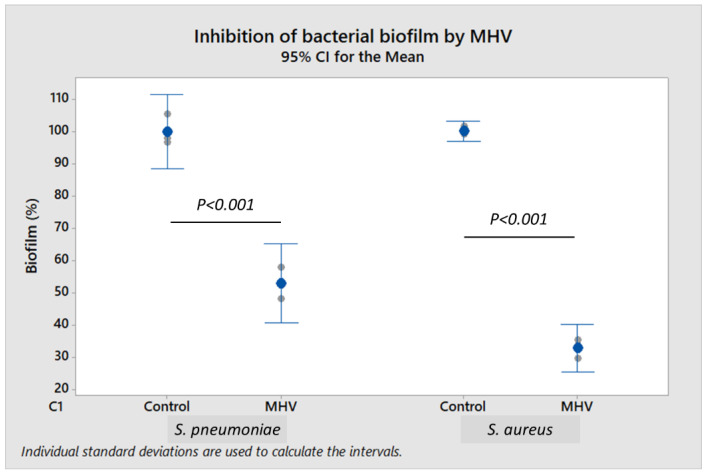
Characterization of biofilm production by *S. pneumoniae* 19F and *S. aureus* A10 strains when co-incubated with murine hepatitis virus (MHV) or cell-free culture supernatant (control) using the crystal violet assay. Each result was derived from three independent experiments or biological replicates (with each assay performed as technical triplicates). Each small gray datapoint represents the average or mean of technical triplicates, while each blue datapoint depicts the mean of three independent experiments. Intervals represent 95% CI for the mean. *p*-value < 0.05 was considered statistically significant by one-way ANOVA with Dunnett’s T3 post-hoc test.

To further demonstrate the effect of live coronavirus on bacterial biofilm formation, murine coronavirus (MHV strain A59) propagated in H2.35 murine liver cells was used as a surrogate for SARS-CoV-2. To study biofilm formation, *S. pneumoniae* and *S. aureus* were incubated respectively with 2.0–4.0 × 10^4^ plaque-forming units (PFU/mL) and 2.0–4.0 × 10^5^ PFU/mL of MHV. When compared to control, *S. pneumoniae* biofilm was reduced by 47.0% (*p* < 0.001), and *S. aureus* biofilm decreased by 67.0% (*p* < 0.001) in the presence of viable MHV (Figure 3). Cell-free culture supernatant of H2.35 cells (without MHV infection), cultured at 35 °C in Dulbecco’s modified Eagle medium (DMEM) supplemented with 10% fetal bovine serum or FBS (in which serum albumin is a major component), served as control. The presence of MHV did not have any significant impact (*p* ≥ 0.05) on the bacterial growth (Supplementary File S4), indicating that the inhibition of biofilm formation by MHV was independent of bacterial load.

The above experiments were carried out using conventional crystal violet assays for semi-quantitative measurements of biofilm formation. However, crystal violet staining does not provide information on the differences between physical and chemical properties of biofilms of different bacteria. Thus, we harnessed particle-tracking microrheology to investigate the viscoelasticity of *S. pneumoniae* and *S. aureus* biofilms. This technique allows measurement of the viscoelastic response in localized regions of the sample, thereby characterizing the heterogeneity within the bacterial biofilm. Formation of a solid-like gel can be easily recognized by the plateauing bead mean square displacement $\langle \Delta x^{2}\left(t\right)\rangle$ at long lag-times *t*.

**Figure 4:**
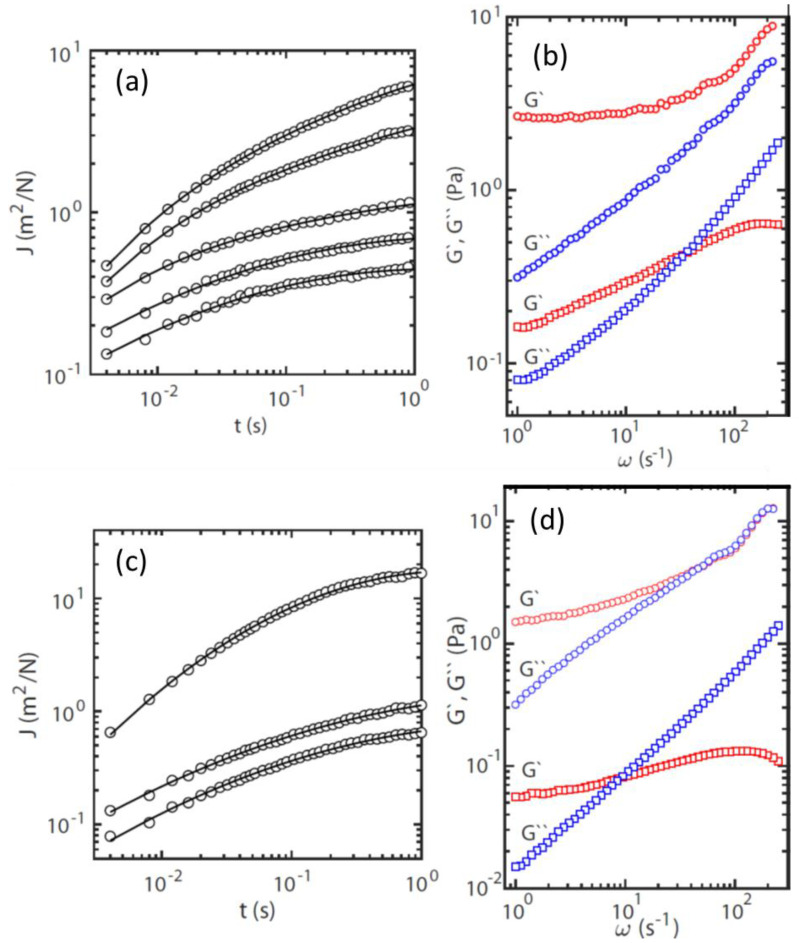
Particle-tracking microrheology analysis of biofilms of (**a**,**b**) *S. pneumoniae* and (**c**,**d**) *S. aureus*. (**a**,**c**) Creep compliance *J* versus lag-time *t* at 296 K corresponding to different beads were measured at randomly chosen locations in each sample. (**b**,**d**) Shown are the elastic storage modulus G′ (red) and viscous loss modulus G″ (blue) versus frequency ω, corresponding to the highest (bottom pair) and lowest (top pair) J(t), respectively.

From the trajectories of the colloidal beads, the probability distributions for displacements in the *x-* and *y*-directions were determined for a range of lag-times *t*. Gaussians were fitted to the probability distributions by optimizing $\langle \Delta x^{2}\left(t\right)\rangle$. The widths of the distributions obtained in the orthogonal *x*- and *y*-directions were averaged. Accordingly, $\langle \Delta x^{2}\left(t\right)\rangle$ refers to the mean square displacement in one dimension. The time-dependent creep compliance of the biofilm can then be derived according to: $J\left(t\right)=3\pi a\;\langle \Delta x^{2}\left(t\right)\rangle /kT$ (with kT being thermal energy, and a the radius of the bead). Figure 4a,c show J(t) at randomly selected locations within the *S. pneumoniae* and *S. aureus* biofilms, respectively. The temporal behavior of J(t) shows that it initially increases and subsequently approaches a plateau at a constant value with increasing lag-time. Furthermore, J(t) depends on the location of the monitored bead inside the sample. For all but one location, the plateau value was not reached within the window of observation. We have verified that the variation of the creep compliance over location within the biofilm is not related to variation in the bead diameter, but to inhomogeneity in the viscoelastic response.

The viscoelastic response of the biofilm is more conveniently discussed in terms of the elastic storage and viscous loss moduli, G′ and G″, respectively. Following the previously described procedure, we have obtained the moduli from the one-sided, complex Fourier transformation of the creep compliance and the generalized Stokes-Einstein equation [18]. This approach requires that the fluid be treated as an incompressible continuum, with no-slip boundary conditions, and that the Stokes drag can be extended over all frequencies [19]. Figure 4b,d present the viscoelastic moduli as a function of frequency, corresponding to the locations in the biofilm with the highest and lowest creep compliances in Figure 4a,c. The moduli pertaining to the other probed locations lie between these two extremes.

The variation of G′ and G″ across the biofilm is a signature of its mechanical inhomogeneity. For the location with the lowest creep compliance, the low frequency limit of G′ is almost constant and corresponds with 1/J(t→∞). Its viscoelastic behavior at the relevant time scale (<1 s) is close to that of an elastic solid. For the other locations, a constant low frequency limiting value of G′ was not observed, which is most likely due to the limited window of observation, with a maximum lag time of 1 s. The value of G′ at the lowest frequency (1 Hz) varies across the biofilm over an order of magnitude in the range 0.2 to 2.5 Pa. The relaxation time τ of the biofilm can be derived from the cross-over frequency of G′ and G″, that is, ω_c_ =1/τ. Again, for some locations, ω_c_ falls outside our window of observation, but it varies in the range 5 to 30 ms, with the shorter relaxation times pertaining to (stiffer) locations with a higher elasticity modulus. The results for the *S. aureus* biofilm are close to those of *S. pneumoniae* biofilm.

Overall, the rheological results indicate that *S. pneumoniae* and *S. aureus* biofilms are elastic and solid-like, with mechanical inhomogeneity related to variations in density of the cross-linking units of their biopolymers (e.g., exopolysaccharides, extracellular DNA, and proteins).

**Figure 5:**
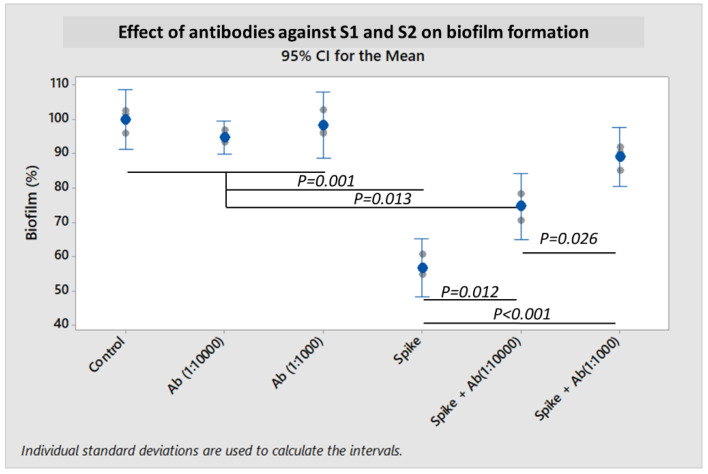
Effect of co-incubating SARS-CoV-2 spike S1 and S2 antibodies with spike S1 + S2 protein on biofilm production by *S. pneumoniae* 19F assessed by the crystal violet assay. The dilutions of both anti-S1 and anti-S2 antibodies used are denoted as 1:10,000 and 1:1000. Spike antibodies (Ab) were co-incubated with SARS-CoV-2 recombinant spike S1 + S2 subunits (10 pmol) for 1 h. Bacteria were then added, and further co-incubated for 18 h for biofilm formation. In the control, no spike antibodies nor proteins were co-incubated with bacteria. Co-incubations of spike antibodies only with bacteria were also tested as controls. Each result was derived from three independent experiments or biological replicates (with each assay performed as technical triplicates). Each small gray datapoint represents the average or mean of technical triplicates, while each blue datapoint depicts the mean of three independent experiments. Intervals represent 95% CI for the mean. *p*-value < 0.05 was taken as statistically significant by one-way ANOVA with Dunnett’s T3 post-hoc test.

To further demonstrate that the SARS-CoV-2 spike protein and its subunits were directly or indirectly responsible for the inhibition of bacterial biofilm formation, experiments were performed by co-incubating antibodies to the S1 subunit and to the S2 ECD. Biofilm formation was partially restored in an antibody concentration-dependent manner (Figure 5). Co-incubation of S1 + S2 subunits together with antibodies at 1:10,000 dilution could rescue *S. pneumoniae* biofilm formation by 17.9% (*p* = 0.012). When the concentrations of antibodies were increased to 1:1000 dilution, *S. pneumoniae* biofilm was further restored by 32.3% (*p* < 0.001).

**Figure 6:**
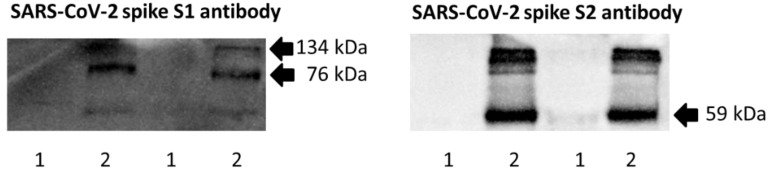
Western blot detection of recombinant SARS-CoV-2 spike S1 + S2 subunits bound to *S. pneumoniae* biofilm (using whole bacterial cell lysate). The blots were individually probed with spike S1 and S2 antibodies. The predicted molecular masses of recombinant spike S1, S2 ECD, and S1 + S2 proteins are 76, 59, and 134 kDa, respectively. Lane 1: Control of *S. pneumoniae* only; lane 2: *S.* *pneumoniae* incubated with S1 + S2 subunits. The results of biological duplicate experiments are shown.

Western blot experiments revealed that SARS-CoV-2 spike protein (i.e., S1 + S2 subunits) was bound to *S. pneumoniae*, and could be detected by antibodies against spike S1 subunit and S2 ECD (Figure 6). In this experiment, bacteria were incubated with S1 + S2 subunits (10 pmol/mL) in 96-well plates for 18 h at 37 °C in an atmosphere of 5% CO_2_. Prior to Western blotting, planktonic and biofilm bacteria were collected and washed with sterile PBS to remove unbound spike protein.

To further investigate the molecular basis of virus–bacteria interactions between the SARS-CoV-2 spike protein or MHV with *S. pneumoniae* or *S. aureus*, untargeted liquid chromatography–mass spectrometry (LC–MS) analysis was performed. A total of 863 proteins were annotated for the resultant dataset of interactions between *S. pneumoniae* and coronavirus (Supplementary File S1). Among these, 731 proteins were annotated for the *S. pneumoniae* control: 687 proteins for *S. pneumoniae* with S1 subunit; 700 proteins for *S. pneumoniae* with S2 ECD; 721 proteins for *S. pneumoniae* with S1 + S2 subunits; 690 proteins for *S. pneumoniae* with MHV. Notably, 87 proteins were differentially annotated for *S. pneumoniae* control: 32 proteins for S1 subunit (including S1 spike protein); 37 proteins for S2 ECD (including S2 spike protein); 36 proteins for S1 + S2 subunits (including S1 and S2 spike proteins); 79 proteins for MHV (inclusive of spike and nucleocapsid proteins). Proteins of interest are presented in Table 2 and Table 3.

In total, 950 proteins were annotated for the dataset of interactions between *S. aureus* and coronavirus. Among these, 820 proteins were annotated for the *S. aureus* control: 772 proteins for *S. aureus* with S1; 813 proteins for *S. aureus* with S2; 791 proteins for *S. aureus* with S1 + S2 subunits; 646 proteins for *S. aureus* with MHV. Notably, 121 proteins were differentially annotated for *S. aureus* control: 51 proteins for S1 (including S1 spike protein); 60 proteins for S2 (including S2 spike protein); 53 proteins for S1 + S2 subunits (including S1 and S2 spike proteins); 33 proteins for MHV. Proteins of interest are presented in Table 2 and Table 3.

## 3. Discussion

Numerous studies indicate that pneumococcal infection is associated with preceding or concomitant virus infections, and that virus infections enhance bacterial growth or dissociation from nasopharyngeal tissue [20]. Influenza A virus (IAV) is associated with greater susceptibility to pneumococcal pneumonia [21,22,23]. Furthermore, IAV infection is linked with increased pneumococcal spread between infant mice, suggesting a role for IAV in the release of *S. pneumoniae* from biofilm and its subsequent transmission [24]. The impact of virus infection on pneumococcal biofilm integrity was demonstrated using a static biofilm model with live cultures of human respiratory epithelial cells that survived with biofilm bacteria. In addition, IAV infection of mice could cause active egress of *S. pneumoniae* from biofilms, and the dispersed bacteria could disseminate in the host to otherwise sterile sites [25]. Similarly, *S. aureus* biofilms grown on the upper respiratory epithelial substratum can disperse in response to host physiologic changes related to viral infection. Mice colonized with *S. aureus* and subsequently exposed to these physiological stimuli or IAV co-infection can develop pronounced pneumonia [26].

The SARS-CoV-2 spike protein is essential for the induction of neutralizing antibody and T-cell responses [27,28]. Importantly, the spike protein is the major target antigen of vaccines against COVID-19, as well as the main site for mutations in SARS-CoV-2 variants, including Omicron [29,30]. Spike expression in human pro-monocytic cells can also dysregulate expression of host genes associated with virus receptor activity, heat shock protein binding, endoplasmic reticulum stress, antigen processing, and presentation [31]. The heavily glycosylated trimeric spike protein consists of two subunits—the S1 subunit contains a receptor-binding domain (RBD) that binds to the host cell receptor (angiotensin-converting enzyme 2 or ACE2), while the S2 subunit mediates fusion between the viral and host cell membranes [32]. During viral infection, the S protein is cleaved by protease into the S1 and S2 subunits. The S1 subunit can induce TLR4 signaling and activate glycolytic metabolism, associated with production of pro-inflammatory cytokines in monocytes/macrophages [33,34]. The pro-inflammatory responses induced by the S1 subunit can contribute to COVID-19-like acute lung injury in Κ18-hACE2 transgenic mice and barrier dysfunction in human endothelial cells [35].

The surface S1 subunit is organized into four domains: an N-terminal domain (NTD), a C-terminal domain (RBD), and two subdomains (SD1 and SD2). The transmembrane S2 subunit contains an N-terminal hydrophobic fusion peptide (FP), two heptad repeats (HR1 and HR2), a transmembrane domain (TM), and a cytoplasmic tail (CT) [36]. Interestingly, both the S1 subunit and S1 + S2 subunits (representing the full-length spike protein) inhibited bacterial biofilm formation—thus indicating that S1 was mainly responsible for this inhibition. In the present study, we demonstrated that murine coronavirus and SARS-CoV-2 spike protein, particularly its S1 subunit, could inhibit or disperse biofilms of both *S. pneumoniae* and *S. aureus* (Figure 2 and Figure 3).

The detection of the SARS-CoV-2 spike protein after bacterial co-incubation (Figure 6) suggests that the interaction between spike protein and *S. pneumoniae* likely involved direct adhesion of the S1 subunit to bacterial surface molecule(s). Antibody concentration-dependent restoration of the biofilm-forming capability of *S. pneumoniae* using target-specific antibodies (Figure 5) confirmed that the spike protein was directly involved in the inhibition or dispersion of *S. pneumoniae* biofilm. Future studies should focus on the identification and function of *S. pneumoniae* surface molecule(s) targeted by SARS-CoV-2 spike protein. Infection of cell lines and differentiated primary human airway cells with NL63 human coronavirus can enhance adherence of *S. pneumoniae* [37]. Thus, it would also be interesting to explore whether SARS-CoV-2 infection or its spike protein can influence pneumococcal adherence to host cells.

Bacterial co-infections and secondary infections occur in COVID-19 patients at an overall proportion of about 7%, and are more common among critically ill patients (8%) [38,39]. *S. pneumoniae* and *S. aureus* (including methicillin-resistant *S. aureus*) represent common Gram-positive pathogens that cause such co-infections [40,41,42,43]. However, it is noteworthy that COVID-19 patients can also be co-infected with Gram-negative bacteria such as *Klebsiella pneumoniae*, *Pseudomonas aeruginosa*, *Escherichia coli,* and *Acinetobacter baumannii* (including antibiotic-resistant strains) [40,41,42,43,44]. Furthermore, SARS-CoV-2 spike protein can bind to lipopolysaccharide (LPS), modulate LPS aggregation, and boost pro-inflammatory activity, culminating in excessive inflammation [45]. Hence, it would be interesting to investigate the interactions between SARS-CoV-2 spike proteins and Gram-negative bacterial biofilms.

The biofilms of *S. pneumoniae* and *S. aureus* are either true elastic soft solids with a yield stress value, or viscoelastic liquids with a finite viscosity. Our microrheology experiments indicate that they are soft solids because: (a) the creep compliance plateaus at a constant value for lag times of around 1 s, and (b) the elastic modulus levels off at a constant value at low frequencies (Figure 4). If the film is a viscoelastic fluid, its relaxation time would be very long, exceeding tens of seconds. The elasticity modulus and relaxation time vary across the biofilms over an order of magnitude, but remain in the order of 1 Pa and 10 ms, respectively. This variation in viscoelastic response is presumably related to inhomogeneity in the density of the cross-linking extracellular biopolymer units, or local segregation (i.e., localized macroscopic inhomogeneity in mass density). The inhomogeneous nature of the samples in this study is consistent with the dynamic characteristics of biofilm development. The viscoelastic responses of the biofilms are close to those for an inhomogeneous soft solid formed by DNA and a nucleoid-associated protein, which indicates a similarity in biomolecular composition [46]. Hart et al. [47] demonstrated that during the initial stages of *S. aureus* biofilm development, column-like structures with a gradient of viscoelasticity are established and modulated by the hydrodynamic shear caused by fluid flow in the environment. In this study, we have demonstrated that particle tracking microrheology can be harnessed to investigate the physical and chemical characteristics of biofilm. Future investigations should focus on the viscoelastic properties of biofilms formed by these bacteria with and without interaction with different spike protein subunits or coronaviruses.

The observed inhibition or dispersion of *S. pneumoniae* and *S. aureus* biofilms was corroborated at the molecular level by untargeted LC-MS-based proteomics (Table 2 and Table 3). Notably, proteins involved in quorum sensing and biofilm formation were upregulated by interaction of the bacteria with SARS-CoV-2 spike protein and MHV. S-ribosylhomocysteine lyase (*luxS*) was upregulated for virus interactions with both *S. aureus* and *S. pneumoniae*. AI-2E family transporter (*yhhT_1*) was also differentially expressed in spike- and MHV-treated *S. pneumoniae*, but not in the control. For biofilm formation, bacteria regulate gene expression in response to changes in their population density through quorum sensing [48]. Quorum sensing is mediated by secreted molecules known as auto-inducers (AIs), which are metabolic by-products of a *luxS* gene-encoded synthase. It is documented that *S. pneumoniae luxS* mutant strain displays low biofilm formation capacity in vitro [49]. Moreover, LuxS plays an important role in *S. pneumoniae* colonization and persistence in the nasopharynx of mice [50]. A murine model of intranasal pneumococcal challenge revealed that the ability to spread from the nasopharynx to the lungs or blood was compromised in a *S. pneumoniae luxS* mutant when compared with the wild-type strain [51]. Similarly, *S.* *aureus* biofilm formation is triggered by AI-2, whose biosynthesis is mediated by LuxS and methylthioadenosine/S-adenosylhomocysteine nucleosidase (MTAN) [52]. However, it is also observed that *luxS* knockout in *S. epidermidis* augments biofilm formation in vitro and enhances virulence in a rat model [53]. These findings indicate that the LuxS quorum-sensing system likely plays key roles in biofilm formation via complex regulatory mechanisms involving many other proteins and molecules [54].

The cell surface of most Gram-positive bacteria contains wall teichoic acid (TA) and lipoteichoic acid (LTA). D-alanine-D-alanyl carrier protein ligase (*dltA*) in *S. pneumoniae* catalyzes the D-alanylation of LTA. Pneumococcal capsule in concert with D-alanylation of LTA contribute to resistance to neutrophil extracellular traps (NETs)-mediated killing in vitro, and promote dissemination to the lungs and bloodstream of mice [55]. Although the role of D-alanylation of LTA in *S. pneumoniae* biofilm development has not been well-established, D-alanyl esters on LTA of *Streptococcus gordonii* are involved in adhesion and biofilm formation [56]. Furthermore, impaired D-alanylation of LTA abrogates the ability of *S. aureus* to colonize any surface and to form antibiotic-resistant biofilms [57].

UDP-N-acetylmuramoyl-L-alanyl-D-glutamate-L-lysine ligase (*murE*) of *S. pneumoniae* catalyzes the addition of L-lysine to the nucleotide precursor UDP-N-acetylmuramoyl-L-alanyl-D-glutamate (UMAG) during the biosynthesis of bacterial cell-wall peptidoglycan. Choline-binding-anchored murein hydrolase (*lytC*) or 1,4-beta-N-acetylmuramidase is a gene encoding one of the three murein hydrolases of *S. pneumoniae*. Sensor protein CiaH is a sensor histidine kinase of *S. pneumoniae*. Mutations in *murE*, *lytC,* and *ciaH* of *S. pneumoniae* impair biofilm formation and nasopharyngeal colonization in mice [58]. In addition, N-acetylneuraminate lyase (*nanA*), a virulence factor and surface enzyme that interacts with host components, is transcriptionally induced during biofilm growth [59]. Interestingly, pneumococcal adhesin, surface protein A (*pspA*), was not differentially expressed with spike and MHV treatment of bacteria—implying the involvement of bacterial cell wall components in *S. pneumoniae* interactions with coronavirus.

The LysR family of transcriptional regulators represents the most abundant type of transcriptional regulator in the prokaryotic kingdom. A putative LysR-type regulator can inhibit biofilm synthesis in *Pseudomonas aeruginosa* [60]. However, the role of LysR transcriptional regulator (*cysB*) in *S. pneumoniae* biofilm formation is unknown.

HTH-type transcriptional regulator MgrA of *S. aureus* negatively regulates biofilm formation and detachment by repressing the expression of phenol-soluble modulin (PSM) operons [61]. PSMs mediate multiple functions during biofilm development and virulence in staphylococcal pathogenesis.

Mature biofilms are composed of bacteria, extracellular polysaccharide, extracellular DNA (eDNA), and proteins. An important virulence factor of *S. aureus* is micrococcal nuclease (MN), a thermostable endonuclease that degrades eDNA as a constituent of the biofilm [62]. Biofilm formation and maturation is expected to be intrinsically affected by the production of MN since it cleaves eDNA. SAOUHSC_00818 in *S. aureus* is predicted to encode a micrococcal nuclease and may affect biofilm maturation.

Although the role of CDP-diacylglycerol-glycerol-3-phosphate 3-phosphatidyltransferase (SAOUHSC_01260) in biofilm formation is unclear, this transmembrane protein catalyzes the committed step to the synthesis of acidic phospholipids, which constitute an important component of the bacterial plasma membrane. The cell wall hydrolase LytN, an autolysin, is probably involved in peptidoglycan hydrolysis. Autolysis is an important process in cell wall turnover in *S. aureus*—mediated by several peptidoglycan hydrolases (or so-called autolysins) and controlled by many regulators. Alterations in autolysin expression (including *lytN*) are suggested to enhance biofilm formation in *S. aureus rot* mutant. The roles of bPH_3 domain-containing protein (SAOUHSC_02568), ribosome maturation factor RimM, and AA_permease domain-containing proteins (SAOUHSC_01803 and SAOUHSC_02590) in *S. aureus* biofilm formation have not been established.

From the results of this in vitro study, we propose that SARS-CoV-2 and related coronavirus infections may trigger an active dispersion of bacteria from biofilm. It has been documented that biofilm bacteria display lower virulence in vivo when compared to broth-grown bacteria [63,64]. Dispersed pneumococci are able to colonize the nasopharynx as well as able to disseminate into the lungs and middle ear to a greater extent than planktonic, broth-grown bacteria and biofilm bacteria [25]. The dispersion of opportunistic pathogens from biofilms during bacterial co-infections or superinfections of COVID-19 patients may explain poorer clinical outcomes, including increased mortality. However, the findings of this in vitro study did not consider host physiologic and immunologic factors. Therefore, future experiments using animal models are essential to firmly establish the putative association between SARS-CoV-2 and bacterial biofilms together with their interacting components.

In conclusion, this in vitro study has provided phenotypic and molecular evidence to enhance our understanding of inter-kingdom interactions between viruses and bacteria. This proof-of-concept study suggests an association between SARS-CoV-2 spike protein and the related murine coronavirus, together with biofilm formation of opportunistic bacterial pathogens *S. pneumoniae* and *S. aureus*. Further detailed investigations are warranted to establish bacterial biofilms as potential risk factors in the development of secondary bacterial pneumonia in COVID-19 patients.

## 4. Materials and Methods

### 4.1. Bacterial Strains

*S. pneumoniae* and *S. aureus* strains initially screened and analyzed in this study are listed in Table 1.

### 4.2. Recombinant SARS-CoV-2 Proteins

Recombinant SARS-CoV-2 spike protein S1 subunit (Val16-Arg685) expressed in HEK293 cells with a poly-histidine tag at the C-terminus (40591-V08H), S2 ECD (Ser686-Pro1213) expressed in baculovirus–insect cells with a poly-histidine tag at the C-terminus (40590-V08B), S1 + S2 subunits (Val16-Pro1213) expressed in baculovirus–insect cells with a poly-histidine tag at the C-terminus (40589-V08B1), and nucleocapsid protein (NP) (Met1-Ala419) expressed in baculovirus–insect cells with a poly-histidine tag at the C-terminus (40588-V08B) were all purchased from Sino Biological (Beijing, China). Lyophilized powder of the recombinant proteins was reconstituted by adding sterile deionized water to prepare a stock solution of 0.25 μg/mL.

### 4.3. SARS-CoV-2 Antibodies

The primary and secondary antibodies (Sino Biological) employed for co-incubation experiments and Western blot analysis were rabbit polyclonal antibody against SARS-CoV-2 spike S1 subunit (40591-T62), and rabbit polyclonal antibody against SARS-CoV-2 spike S2 (40590-T62). The goat anti-rabbit IgG-Fc antibody conjugated with HRP reporter (SSA003) served as the secondary antibody for Western blotting.

### 4.4. Growing S. pneumoniae Biofilm

*S. pneumoniae strains* were grown on BBL trypticase soy agar with 5% sheep blood (TSA II) plates (Becton Dickinson, Franklin Lakes, NJ, USA). Plates were incubated for 18–24 h at 37 °C with 5% CO_2_. A single bacterial colony was inoculated into 10 mL of sterile BHI broth (Sigma-Aldrich, St Louis, MO, USA), 10-fold serially diluted into the same medium, and propagated overnight for 18 h in an atmosphere of 5% CO_2_. Overnight cultures that were still in exponential phase (OD600 ≈ 0.2–0.4) were selected for experiments. For growing *S. pneumoniae* biofilm, 1 mL of overnight culture was inoculated into 9 mL of fresh BHI broth, and incubated at 37 °C with 5% CO_2_ until OD600 ≈ 0.4 was reached. When *S. pneumoniae* culture reached 2.0–4.0 × 10^8^ CFU/mL, the culture was diluted 10-fold with fresh BHI broth, and 100 μL of diluted culture was inoculated into each well of a sterile Cellstar 96-well plate (Greiner Bio-One, Frickenhausen, Germany) containing 100 μL of BHI with 2% *D*-glucose (1st BASE, Singapore). The final concentration of *S. pneumoniae* was 2.0–4.0 × 10^6^ CFU per well. The 96-well plate was incubated at 37 °C for 18 h in an atmosphere of 5% CO_2_.

### 4.5. Growing S. aureus Biofilm

*S. aureus* strains were grown on nutrient agar plates (Oxoid, Basingstoke, England, UK) at 37 °C for 18–24 h. A single bacterial colony was inoculated into 10 mL of sterile nutrient broth and propagated overnight for 18 h. For growing *S. aureus* biofilm, 1 mL of overnight culture was inoculated into 9 mL of fresh nutrient broth, and incubated at 37 °C until OD600 ≈ 0.4 was reached. When *S. aureus* culture reached 2.0–4.0 × 10^8^ CFU/mL, 100 μL of culture was inoculated into each well of a sterile Cellstar 96-well plate containing 100 μL of nutrient broth with 2% *D*-glucose. The final concentration of *S. aureus* was 2.0–4.0 × 10^7^ CFU per well. The 96-well plate was incubated at 37 °C for 18 h.

### 4.6. Crystal Violet Assay for Biofilm Estimation

After 18 h of incubation, expended medium was carefully aspirated from the wells and discarded. The wells were washed thrice each with 200 μL of sterile phosphate-buffered saline (PBS). The wells were air-dried and stained for 15 min with 200 μL of 0.1% crystal violet (dissolved in water). Excess crystal violet was aspirated from the wells, which were washed thrice each with 200 μL of sterile distilled water. Adherent crystal violet was dissolved by adding 200 μL of 95% ethanol into each well. The dissolved crystal violet solution was transferred to a new microplate and optical density (at 570 nm) was measured using an Infinite M200 Plate Reader (Tecan, Mannedorf, Switzerland). To compensate for background absorbance, readings were subtracted from the average value of sterile medium and crystal violet (reference blank).

### 4.7. Co-Incubation of Spike Proteins with Bacterial Biofilms

Recombinant spike protein stocks were diluted to 10 pmol/mL with BHI or nutrient broth with 2% *D*-glucose—each solution (100 μL) was dispensed into their respective wells of a 96-well plate. When *S. pneumoniae* culture reached 2.0–4.0 × 10^8^ CFU/mL, the culture was diluted 10-fold with fresh BHI broth, and 100 μL of diluted culture was inoculated into each well. The 96-well plate was incubated at 37 °C in an atmosphere of 5% CO_2_. Similarly, when *S. aureus* culture reached 2.0–4.0 × 10^8^ CFU/mL, 100 μL of culture was inoculated into each well of a 96-well plate, and incubated at 37 °C. After 18 h of incubation, the biofilm was estimated by crystal violet assay.

### 4.8. Co-Incubation of Spike Proteins and Antibodies with Bacterial Biofilms

Recombinant spike protein stocks and antibodies were diluted to the desired concentrations with BHI or nutrient broth with 2% *D*-glucose. Subsequently, each solution (100 μL) was dispensed into their respective wells of a 96-well plate, and incubated at room temperature for 1 h with constant shaking. When *S. pneumoniae* culture reached 2.0–4.0 × 10^8^ CFU/mL, the culture was diluted 10-fold with fresh BHI broth, and 100 μL of diluted culture was inoculated into each well. The 96-well plate was incubated at 37 °C in an atmosphere of 5% CO_2_. Similarly, when *S. aureus* culture reached 2.0–4.0 × 10^8^ CFU/mL, 100 μL of culture was inoculated into each well of a 96-well plate, and incubated at 37 °C. After 18 h of incubation, the biofilm was estimated by crystal violet assay.

### 4.9. Co-Incubation of Mouse Hepatitis Virus (MHV) with Bacterial Biofilm

MHV A59 strain was diluted to the desired concentrations (2.0–4.0 × 10^4^ PFU/mL for *S. pneumoniae*, and 2.0–4.0 × 10^5^ PFU/mL for *S. aureus*) with BHI or nutrient broth with 2% *D*-glucose. Then, MHV suspension (100 μL) was dispensed into their respective wells of a 96-well plate. When *S. pneumoniae* culture reached 2.0–4.0 × 10^8^ CFU/mL, the culture was diluted 10-fold with fresh BHI broth, and 100 μL of diluted culture inoculated into each well. The 96-well plate was incubated at 37 °C in an atmosphere of 5% CO_2_. Similarly, when *S. aureus* culture reached 2.0–4.0 × 10^8^ CFU/mL, 100 μL of culture was inoculated into each well. This resulted in virus PFU-to-bacteria CFU ratios of 0.001. The 96-well plate was incubated at 37 °C for 18 h, and the biofilm was estimated by crystal violet assay.

### 4.10. Passive Microrheology (Microparticle Video Tracking) of Biofilm

The biofilms were cultured in the presence of polystyrene microspheres (Polysciences, Warrington, PA, USA), each of which being 1.93 ± 0.05 µm in diameter. When *S. pneumoniae* culture reached 2.0–4.0 × 10^8^ CFU/mL, the culture was diluted 10-fold with fresh BHI broth, and 100 μL of diluted culture was inoculated together with polystyrene microspheres into each well. The 96-well plate was incubated at 37 °C in an atmosphere of 5% CO_2_. Similarly, when *S. aureus* culture reached 2.0–4.0 × 10^8^ CFU/mL, 100 μL of culture was inoculated together with polystyrene microspheres into each well. The bacterial culture and microspheres were mixed well using a pipette. The 96-well plate was incubated at 37 °C for 18 h. Particle-tracking experiments were then performed with a Nikon Eclipse Ti-U microscope equipped with 100× long working distance objective. The trajectories of different beads in randomly chosen locations, separated by around 100 μm, were recorded with a metal oxide semiconductor (CMOS) camera (Basler A504k) at a rate of 250 frames per second. Video clips of 3–5 minutes’ duration were analyzed with MATLAB (Natick, MA, USA), and the particle trajectories were derived using public domain tracking software at http://site.physics.georgetown.edu/matlab/ (last accessed on 15 November 2021). All further data analysis was carried out using in-house-developed software scripts written in MATLAB code. The pixel resolution of 0.12 µm was calibrated using a metric ruler.

### 4.11. Western Blot Detection of Spike Proteins Binding to Bacteria

Overnight cultures of *S. pneumoniae* and *S. aureus* were harvested, washed, and resuspended in the corresponding media. The bacteria (2.0–4.0 × 10^9^ CFU/mL) were incubated with 10 pmol of spike protein at 4 °C for 90 min. Subsequently, the bacterial suspensions were washed thrice each with sterile PBS, and lysed in RIPA buffer (Thermo Fisher Scientific, Waltham, MA, USA) supplemented with protease and phosphatase inhibitor mini tablets (Pierce; one tablet per 10 mL of RIPA buffer). Protein quantification was performed using the Pierce BCA protein assay kit (Thermo Fisher Scientific), with BSA being used to construct the standard curve. Laemmli buffer (6×) was added to 30 µg of purified protein lysates, to a final concentration of 1×. The protein samples were placed in a dry bath at 100 °C for 5 min, and then centrifuged at 10,000 rpm for 5 min. The purified protein lysates were loaded onto a SDS-PAGE (10%) gel for separation. The separated proteins were then transferred onto a PVDF membrane in transfer buffer at 250 mA for 1 h. The membrane was blocked in 5% BSA, prepared in 0.1% PBST overnight at 4 °C, followed by incubation with primary antibody (1:1000 dilution) in 5% BSA in 0.1% PBST for 1 h at room temperature. The blots were washed thrice in 0.1% PBST for 5 min each, and subsequently incubated with goat anti-rabbit IgG-Fc secondary antibody (1:1000 dilution) for 1 h. The blots were washed thrice in 0.1% PBST for 5 min each, and then incubated in WesternBright Enhanced Chemiluminiscent HRP substrate (Advansta, San Jose, CA, USA) prior to imaging using the ChemiDoc XRS imager (Bio-Rad, Hercules, CA, USA).

### 4.12. Liquid Chromatography-Mass Spectrometry (LC-MS)

Proteins from bacterial samples were extracted in 100 mM triethylammonium bicarbonate (TEAB) buffer (Sigma-Aldrich). For in-trap digestion, protein samples were extracted and processed using the S-Trap micro column (Protifi, Farmingdale, NY, USA) according to the manufacturer’s recommendations. Tryptic peptides were subjected to LC–MS analysis using an Eksigent nanoLC-Ultra and ChiPLC-nanoflex in trap-elute configuration, with a Trajan ProteoCol C18P (3 μm particle size, 120 Å pore size, 300 μm ID × 10 mm length) trap column, and a Thermo Scientific Acclaim PepMap 100 C18 (3 μm particle size, 100 Å pore size, 75 μm ID × 250 mm length) analytical column. Peptides were separated by a gradient formed by 2% acetonitrile, 0.1% formic acid (mobile phase A) and 98% acetonitrile, 0.1% formic acid (mobile phase B): 5–12% B (0–20 min), 12–30% B (20–60 min), 30–90% B (60–62 min), 90–90% B (62–69 min), 90–5% B (69–72 min), and 5–5% B (72–85 min), with the total flow rate maintained at 300 nL/min. The mass spectrometry was performed on a SCIEX TripleTOF 5600 system (nebulizer gas GSI: 12 units, curtain gas: 30 units, IonSpray voltage floating: 2300 V, interface heater temperature: 150 °C) in information-dependent (IDA) mode to switch automatically between MS and MS/MS acquisition. MS spectra were acquired across the mass range of 350–1250 m/z in high-resolution mode using 250 ms accumulation time per spectrum. A maximum of 50 precursors per cycle with the most intense m/z values, with a threshold of >125 counts per second (cps) and charge state between 2+ and 5+, were selected for fragmentation from each MS spectrum. Tandem mass spectra were recorded at 100–1800 m/z in high-sensitivity mode using 50 ms accumulation time, 12 s dynamic exclusion, and with rolling collision energy enabled.

Peptide identification and the detection of post-translational modifications were achieved using the ProteinPilot 5.0 software (AB SCIEX, Framingham, MA, USA) against Uniprot reference proteome databases UP000437160 (for *S. pneumoniae*), UP000008816 (for *S. aureus*), UP000464024 (for SARS-CoV-2), and UP000007192 (for MHV A59); they were spiked with common contaminant proteins (cRAP) using the following parameters: cysteine alkylation—methyl methanethiosulfonate (MMTS); digestion—trypsin; ID focus—biological modifications and amino acid substitutions; search effort—thorough ID. FDR analysis was carried out with detected protein threshold (Unused ProtScore) set at 0.05. Automatic bias correction was selected for calculation of protein ratios. Proteins corresponding to local FDR < 5% and global FDR < 1% were selected for further analyses.

For the estimation of protein concentration, Mascot Server 2.7 (Matrix Science, Boston, MA, USA) was used. A search using the above-mentioned Uniprot reference proteosome databases was carried out using the following parameters: cysteine alkylation—MMTS; fixed modification—methylthio (C); variable modifications—acetyl (protein N-term); oxidation (M); peptide mass tolerance of 100 ppm; fragment mass tolerance of 0.04 Da. The concentrations of detected proteins were estimated by a two-step process. Firstly, the weighted fraction (percentage) of each protein was determined by its exponentially modified protein abundance index (emPAI) score in MS profile and its molecular weight [65]. The weight fraction was then multiplied by the total concentration of the respective sample. Protein content (percentage) was calculated using the following formulas.

MWxEmpai=emPAI×Mass

$$Protein\;content\;\left(\%\right)=\frac{MWxEmpai}{Sum\;of\;MWxEmpai}\times 100$$

## Figures and Tables

**Table 1:** Bacterial strains initially screened and analyzed in this study.

Bacterial Species	Subspecies/Serotype/Strain	Notes	Reference
*S. pneumoniae*	(Klein) Chester (ATCC 49619)		
	Serotype 3 (A66.1 Xen 10)		Moorthy et al., 2016 [17]
	Serotype 4 (TIGR)		Moorthy et al., 2016 [17]
	Serotype 19F	A clinical isolate from Singapore	Moorthy et al., 2016 [17]
	Serotype 2	D39 wild-type strain	Provided by Dr. L.-T. Sham
	Serotype 19A	A clinical isolate from National University Hospital, Singapore	Provided by Dr. L.-T. Sham
*S. aureus*	Subsp. *aureus* Rosenbach (ATCC 25923)		
	Subsp. *aureus* Rosenbach (ATCC 49775)		
	A10	A human nasal isolate	

**Table 2:** Proteins of interest detected by LC-MS for proteomic analyses of bacterial and coronavirus interactions.

	*S. pneumoniae*					*S. aureus*				
Protein/Gene	Control	S1	S2	S1 + S2	MHV	Control	S1	S2	S1 + S2	MHV
ABC transporter substrate-binding proteins (*yclQ* and ERS019258_00662)	N	D	D	D	D	-	-	-	-	-
AI-2E family transporter (*yhhT_1*)	N	D	D	D	D	-	-	-	-	-
D-alanine-D-alanyl carrier protein ligase (*dltA*)	N	D	N	D	D	-	-	-	-	-
Histidine kinase (*ciaH*, *hk08*, *phoR*)	N	D	D	D	D	-	-	-	-	-
LysR family transcriptional regulator (*cysB*)	N	N	N	D	D	-	-	-	-	-
UDP-N-acetylmuramoyl-L-alanyl-D-glutamate-L-lysine ligase (*murE*)	N	D	N	D	N	-	-	-	-	-
AA_permease domain-containing proteins (SAOUHSC_01803 and SAOUHSC_02590)	-	-	-	-	-	D	N	N	N	N
bPH_3 domain-containing protein (SAOUHSC_02568)	-	-	-	-	-	N	D	N	D	N
CDP-diacylglycerol-glycerol-3-phosphate 3-phosphatidyltransferase (SAOU-HSC_01260)	-	-	-	-	-	N	D	N	D	N
Micrococcal nuclease (SAOUHSC_00818)	-	-	-	-	-	N	D	N	D	N
Probable cell wall hydrolase LytN	-	-	-	-	-	N	D	N	D	N
Ribosome maturation factor RimM	-	-	-	-	-	N	D	N	D	N
Uncharacterized protein (SAOUHSC_01627)	-	-	-	-	-	N	D	N	D	D

N: Not detected; D: Detected. Full lists of differentially annotated proteins in Supplementary File S2.

**Table 3:** Differentially expressed proteins with their indicated fold change relative to the bacterial control. The fold change of each annotated protein was calculated based on protein content (percentage) estimated from the exponentially modified protein abundance index (emPAI).

	Fold Change (Times)							
	*S. pneumoniae*				*S. aureus*			
Protein/Gene	S1	S2	S1 + S2	MHV	S1	S2	S1 + S2	MHV
S-ribosylhomocysteine lyase (*luxS*)	0.5	NC	2.8	0.5	4.2	3.7	1.8	3.7
Choline binding-anchored murein hydrolase (*lytC*)	2.3	1.9	2.2	0.6	-	-	-	-
N-acetylneuraminate lyase (*nanA_2*)	0.3	0.7	1.5	0.7	-	-	-	-
Pneumococcal surface protein A (*pspA*)	NC	NC	NC	NC	-	-	-	-
HTH-type transcriptional regulator MgrA (*mgrA*)	-	-	-	-	1.5	0.1	1.9	1.4

NC: No change (0.6 < fold change < 1.5). Full lists of differentially expressed proteins in Supplementary File S3.

## Data Availability

The mass spectrometry proteomics data have been deposited to the ProteomeXchange Consortium via the PRIDE partner repository with the dataset identifier PXD029967.

## Supplementary Materials

The following are available online at https://www.mdpi.com/article/10.3390/ijms23063291/s1.
